# The Destabilized Artery: A Case of Spontaneous Coronary Artery Dissection Presenting as Unstable Angina

**DOI:** 10.7759/cureus.20705

**Published:** 2021-12-26

**Authors:** Ebubechukwu Ezeh, Esiemoghie J Akhigbe, Olusola Olubowale, Onyinye Ugonabo, Mackenzie Hamilton, Benjamin Dao, Jason Mader

**Affiliations:** 1 Internal Medicine, Marshall University Joan C. Edwards School of Medicine, Huntington, USA; 2 Cardiology, Marshall University Joan C. Edwards School of Medicine, Huntington, USA; 3 Cardiology, Marshall University, Huntington, USA

**Keywords:** electrocardiogram (ecg/ekg), troponin, cardiovascular, left heart catheterization, acute coronary syndrome, coronary artery angiography, unstable angina, spontaneous coronary artery thrombosis

## Abstract

Spontaneous coronary artery dissection (SCAD) is increasingly being recognized as a cause of acute coronary syndrome (ACS). This increased recognition of SCAD has been noted in patients with and without traditional cardiovascular risk factors such as diabetes mellitus, hyperlipidemia, and cigarette smoking. The increasing incidence is believed to be due to recent advances in diagnostic and coronary imaging modalities. The most common presenting feature is chest pain or discomfort. Normal troponin level does not rule out SCAD as the definitive diagnosis is made on coronary angiography. Percutaneous intervention (PCI) for SCAD has been associated with lower success rates compared to PCI for atherosclerotic coronary artery disease. Medical management is, therefore, the initial treatment of choice.

## Introduction

Spontaneous coronary artery dissection (SCAD) has gained increased recognition in the last decade, predominantly because of increased awareness and advances in imaging modalities in the cardiac catheterization lab [1]. It has been defined as a nontraumatic and non-iatrogenic separation of the coronary arterial wall by intramural hemorrhage creating a false lumen, with or without an intimal tear [2,3]. SCAD is now estimated to be the cause of approximately 1-4% of acute coronary syndrome (ACS) cases in the general population [1]. The predominant mechanism of myocardial injury occurring as a result of SCAD is coronary artery obstruction caused by the formation of an intramural hematoma (IMH) or intimal disruption rather than atherosclerotic plaque rupture or intraluminal thrombus [4]. Over 90% of patients with SCAD present with chest pain/discomfort, nausea/vomiting, and dyspnea are reported by 20% and 18% of patients respectively [1]. For most patients, extreme physical or emotional stress precipitates the symptoms [4]. In this case report, we present a 66-year-old male who presented with exertional chest pain and was found to have SCAD of the distal left anterior descending artery (LAD).

## Case presentation

A 66-year-old man with a past medical history of hypertension, diabetes mellitus and hyperlipidemia, was referred from an outside facility on account of chest pain. He initially presented to the outside facility with complaints of one episode of chest pain that started 30 minutes prior. Chest pain was described as sharp, mid-sternal, 8 out of 10 in intensity, and non-radiating. It was exacerbated by exertion and had no known relieving factor. He denied any associated nausea, vomiting, fever, palpitations, dyspnea, or vomiting. He reported no prior known coronary artery disease. Initial work-up at the outside facility documented sinus rhythm with non-specific T wave abnormalities on electrocardiogram, and normal troponin levels. D-dimer was elevated, and a chest computed tomography ruled out pulmonary embolism. Chest pain failed to resolve with medical management including nitroglycerin, metoprolol tartrate and heparin, hence warranting transfer to our facility for further workup and possible invasive intervention.

**Figure 1:**
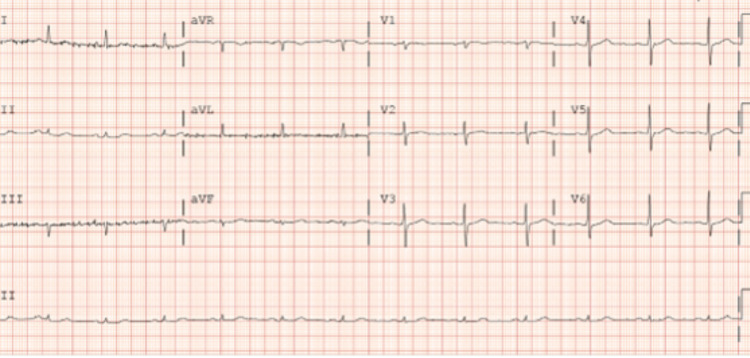
ECG showing NSR with non-specific ST-T wave abnormalities and low voltage complexes on frontal leads NSR: Normal sinus rhythm

**Figure 2:**
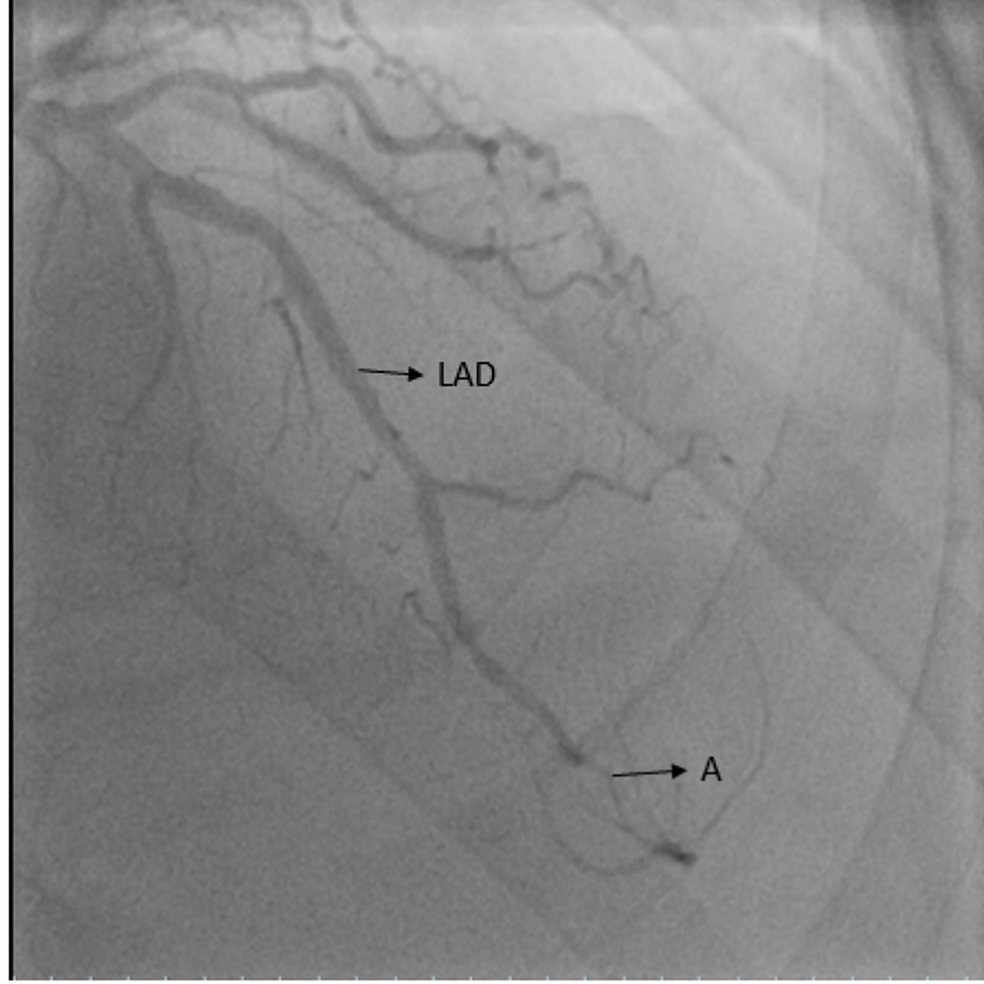
Coronary angiogram showing LAD (left anterior descending artery) and area of dissection (labeled as 'A')

On arrival to our facility, his vital signs were blood pressure 132/76 mmHg, pulse rate 78 beats/minute, and was still having active chest pain. EKG showed normal sinus rhythm (NSR) with non-specific ST/T wave abnormalities and low voltage complexes on frontal leads (Figure 1). There was a suspicion for acute coronary syndrome, hence the patient was taken for urgent ischemic evaluation with left heart catheterization (LHC). LHC showed non-obstructive coronary artery disease (CAD) in the proximal-distal left anterior descending artery (LAD); 30-40%, luminal irregularities in the left circumflex artery, and 20% proximal right coronary artery disease. A type 3 SCAD was noted in the LAD (Figure 2). The distal vessel was small-caliber and there was a concern for the propagation of dissection, hence intra-coronary imaging modalities were not performed. The Troponin trend remained negative throughout his admission, and the echocardiogram showed normal ejection fraction (EF) (62% Bi-plane method) with grade 1 diastolic dysfunction. He was medically managed as unstable angina and SCAD with dual anti-platelets, high-intensity statin, angiotensin-converting enzyme inhibitors, and beta-blocker therapy. The patient remained chest pain-free and hemodynamically stable on medical therapy, and no further intervention was performed. The patient was discharged to a cardiac rehabilitation program and recommended to follow up with the cardiology clinic as an outpatient.

## Discussion

**Figure 3:**
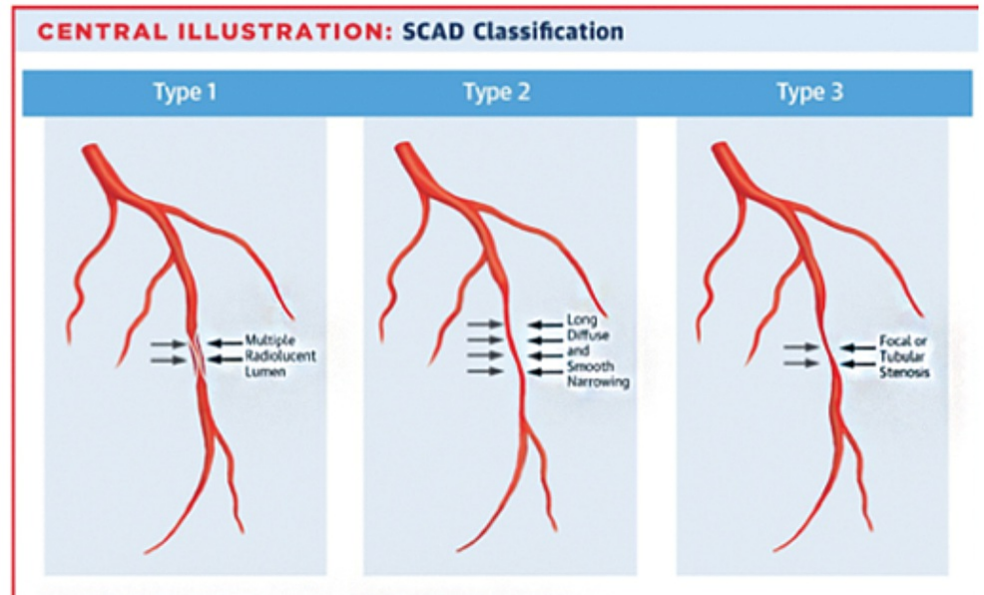
Saw classification of spontaneous coronary artery dissection (SCAD)

SCAD can occur in any coronary artery, but the LAD and its branches are mostly involved in up to 32% to 46% of cases [5,6]. Our patient had SCAD affecting his distal LAD. The Saw angiographic classification categorizes SCAD into three types (Figure 3).

Type 1 refers to the classic appearance of multiple radiolucent lumens [4]. Type 2 refers to the presence of diffuse stenosis that can be of varying severity and length (usually >20 mm; Variant 2A is diffuse arterial narrowing bordered by normal segments proximal and distal to the IMH, and variant 2B is diffuse narrowing that extends to the distal tip of the artery) [4]. Type 3 is focal or tubular stenosis, usually <20 mm in length, that mimics atherosclerosis [4]. Intracoronary imaging is recommended to confirm the presence of IMH and to diagnose SCAD [4]. A number of studies have reported type 2 as the most common angiographic manifestation of SCAD [4,7,8]. Our patient had type 3 dissection.

SCAD has often been described as affecting patients with few or no traditional cardiovascular risk factors. Recent studies have identified that a portion of SCAD patients has traditional risk factors [1]. Though it has a predilection for young women without cardiovascular risk factors, SCAD should still be entertained as a differential diagnosis in men who present with ACS regardless of the presence or absence of cardiovascular risk factors [3]. Our patient had hypertension, hyperlipidemia, and diabetes mellitus. Moreover, a high index of suspicion should be maintained even with normal troponin levels, as up to 27% of patients ultimately diagnosed with SCAD have normal initial troponin levels [5]. This was the case with our patient.

Early and accurate diagnosis of SCAD is key because the management of SCAD significantly differs from that of atherosclerotic disease [9]. In contrast to the approach for patients with myocardial infarction caused by atherosclerosis, the favored approach for patients with SCAD who are in clinically stable condition is aggressive medical treatment. More than 80% of patients can be successfully treated medically [5]. Percutaneous coronary intervention (PCI) for treatment of SCAD has limited success rates and higher rates of complications when compared with PCI for atherosclerotic CAD [10]. Iatrogenic dissection is actually the complication in PCI for SCAD cases [7,10,11].

## Conclusions

SCAD is an important and often, under-diagnosed cause of ACS. Following advances in diagnostic coronary angiography, our understanding of SCAD has significantly increased in recent years. A high index of suspicion should be maintained for diagnosis of SCAD in patients with the typical presentation even in the presence of cardiovascular risk factors. Medical management remains the initial treatment of choice as PCI is associated with worse outcomes.
